# The genome sequence of the Chequered Fruit-tree Tortrix, *Pandemis corylana *(Fabricius, 1794)

**DOI:** 10.12688/wellcomeopenres.20572.1

**Published:** 2024-01-08

**Authors:** Douglas Boyes, Ian Sims, David C. Lees, Marianne Eagles, University of Oxford and Wytham Woods Genome Acquisition Lab, Natural History Museum Genome Acquisition Lab, Darwin Tree of Life Barcoding collective, Wellcome Sanger Institute Tree of Life Management, Samples and Laboratory team, Wellcome Sanger Institute Scientific Operations: Sequencing Operations, Wellcome Sanger Institute Tree of Life Core Informatics team, Tree of Life Core Informatics collective, Darwin Tree of Life Consortium

**Affiliations:** 1 UK Centre for Ecology & Hydrology, Wallingford, England, UK; 2 Syngenta International Research Station, Jealott’s Hill, England, UK; 3 Natural History Museum, London, England, UK; 4 Independent researcher, Crawley Down, England, UK

**Keywords:** Pandemis corylana, Chequered Fruit-tree Tortrix, genome sequence, chromosomal, Lepidoptera

## Abstract

We present a genome assembly from an individual male *Pandemis corylana* (the Chequered Fruit-tree Tortrix; Arthropoda; Insecta; Lepidoptera; Tortricidae). The genome sequence is 441.6 megabases in span. Most of the assembly is scaffolded into 30 chromosomal pseudomolecules, including the Z sex chromosome. The mitochondrial genome has also been assembled and is 15.53 kilobases in length. Gene annotation of this assembly on Ensembl identified 19,608 protein coding genes.

## Species taxonomy

Eukaryota; Metazoa; Eumetazoa; Bilateria; Protostomia; Ecdysozoa; Panarthropoda; Arthropoda; Mandibulata; Pancrustacea; Hexapoda; Insecta; Dicondylia; Pterygota; Neoptera; Endopterygota; Amphiesmenoptera; Lepidoptera; Glossata; Neolepidoptera; Heteroneura; Ditrysia; Apoditrysia; Tortricoidea; Tortricidae; Tortricinae; Archipini; *Pandemis*; *Pandemis corylana* (Fabricius, 1794) (NCBI:txid1101029).

## Background

A common micro-moth of the family Tortricidae, the *Pandemis corylana* is noted for its net-like pattern or reticulation to the yellow and reddish-brown and dark cross banding of the forewing. Found in most of England and Wales and local in Scotland and Ireland, the single-brooded, *Pandemis corylana* is on the wing from July to September, occasionally to October. Its larvae feed on trees and shrubs from May to July in deciduous woodland, scrub, hedgerows and gardens and may be found in spun or folded leaves of hazel, ash, oak, bramble and honeysuckle ( Sterling *et al.*, 2012).

There are five similar *Pandemis* species in the UK, with identification assisted by raising of larvae, and dissection of adults ( British Lepidoptera, 2023; Kimber, 2023). Worldwide there are multiple species of *Pandemi*s, including some important pests on apple. Dombroskie and Sperling (2012) highlighted the importance of a combined approach, using DNA, morphological and geographic evidence to successfully separate similar species where no single source was sufficient. This is an example of areas of work where the completed genome sequence will provide additional evidence.

We present a chromosomally complete genome sequence for *Pandemis corylana* based on one male specimen from Wytham Woods, Oxfordshire, UK, as part of the Darwin Tree of Life Project. This project is a collaborative effort to sequence all named eukaryotic species in the Atlantic Archipelago of Britain and Ireland.

## Genome sequence report

**Figure 1.**
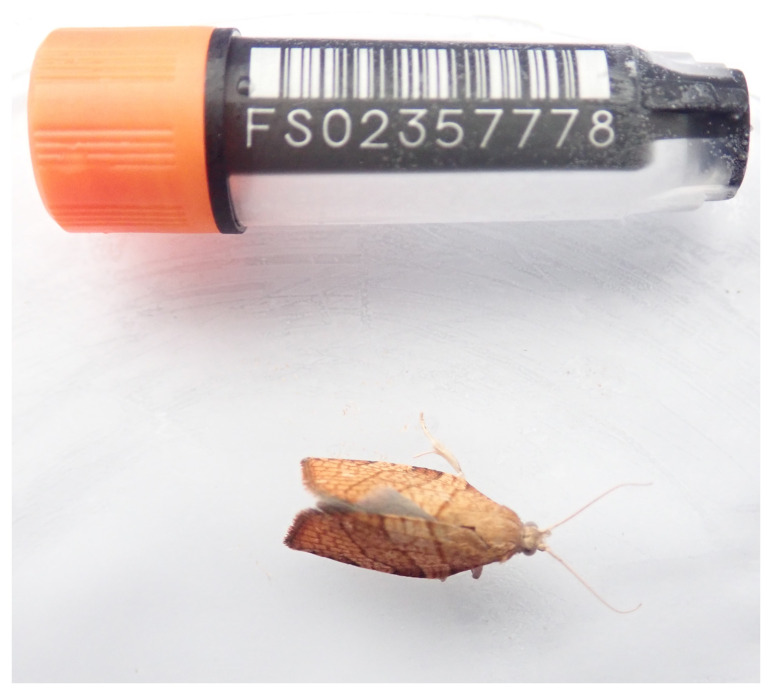
Photograph of the *Pandemis corylana* (ilPanCory1) specimen used for genome sequencing.

The genome was sequenced from one male *Pandemis corylana* ( Figure 1) collected from Wytham Woods, Oxfordshire, UK (51.77, –1.34). A total of 47-fold coverage in Pacific Biosciences single-molecule HiFi long reads was generated. Primary assembly contigs were scaffolded with chromosome conformation Hi-C data. Manual assembly curation corrected 32 missing joins or mis-joins and removed 10 haplotypic duplications, reducing the scaffold number by 2.44%.

**Figure 2.**
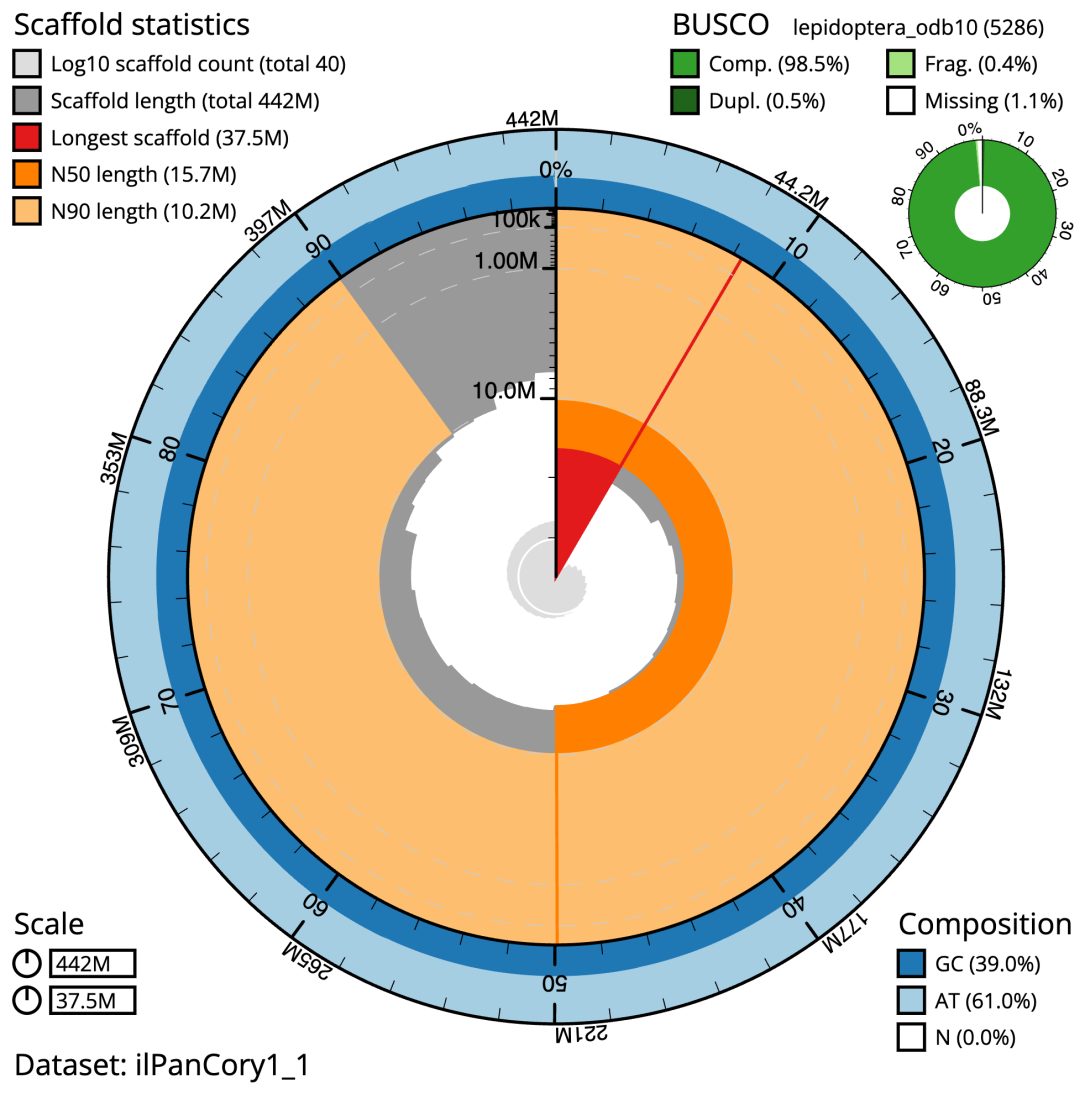
Genome assembly of *Pandemis corylana*, ilPanCory1.1: metrics. The BlobToolKit Snailplot shows N50 metrics and BUSCO gene completeness. The main plot is divided into 1,000 size-ordered bins around the circumference with each bin representing 0.1% of the 441,605,538 bp assembly. The distribution of scaffold lengths is shown in dark grey with the plot radius scaled to the longest scaffold present in the assembly (37,479,866 bp, shown in red). Orange and pale-orange arcs show the N50 and N90 scaffold lengths (15,684,937 and 10,157,545 bp), respectively. The pale grey spiral shows the cumulative scaffold count on a log scale with white scale lines showing successive orders of magnitude. The blue and pale-blue area around the outside of the plot shows the distribution of GC, AT and N percentages in the same bins as the inner plot. A summary of complete, fragmented, duplicated and missing BUSCO genes in the lepidoptera_odb10 set is shown in the top right. An interactive version of this figure is available at https://blobtoolkit.genomehubs.org/view/Pandemis%20corylana/dataset/ilPanCory1_1/snail.

**Figure 3.**
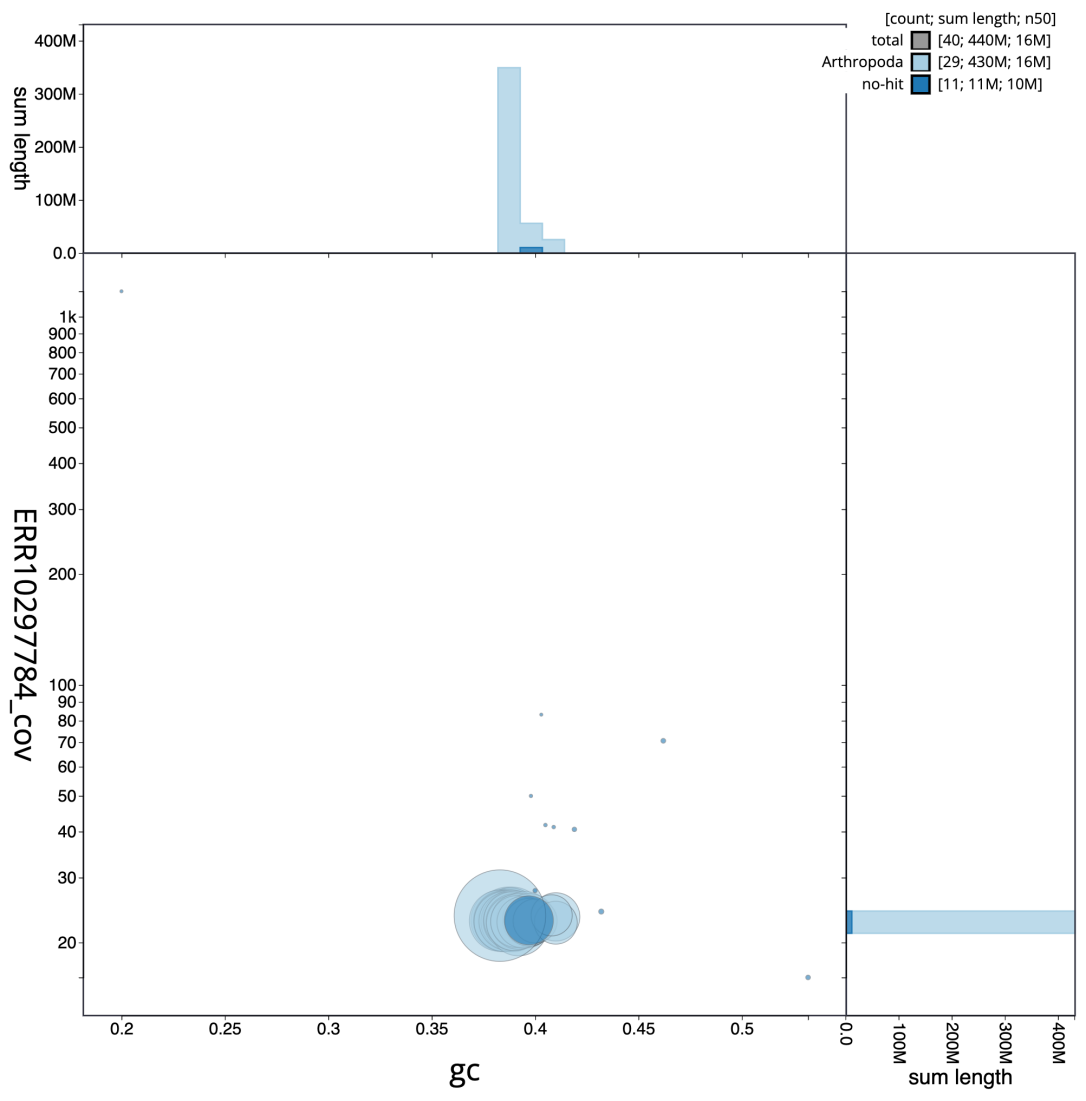
Genome assembly of *Pandemis corylana*, ilPanCory1.1: BlobToolKit GC-coverage plot. Scaffolds are coloured by phylum. Circles are sized in proportion to scaffold length. Histograms show the distribution of scaffold length sum along each axis. An interactive version of this figure is available at https://blobtoolkit.genomehubs.org/view/Pandemis%20corylana/dataset/ilPanCory1_1/blob.

**Figure 4.**
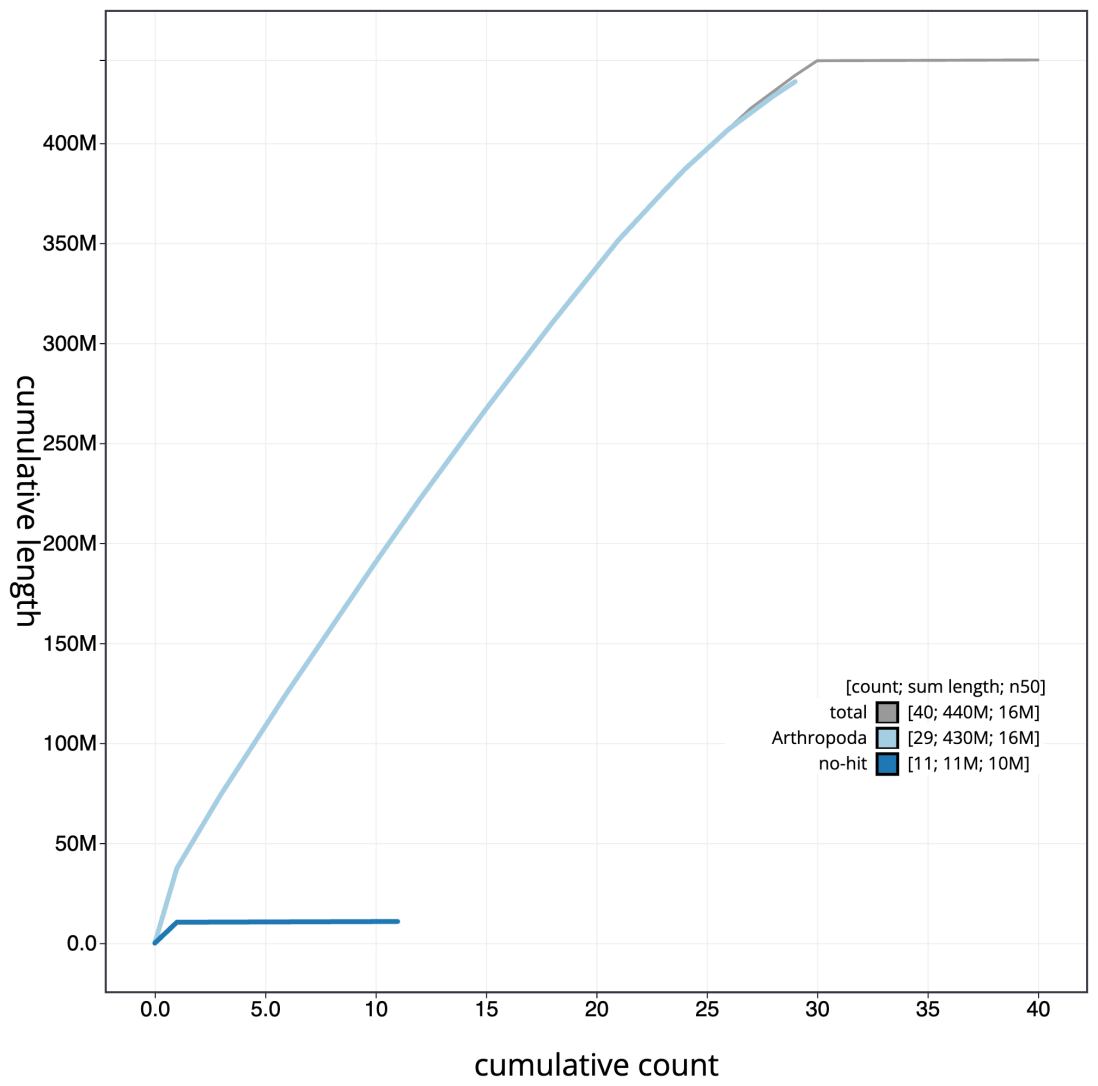
Genome assembly of *Pandemis corylana*, ilPanCory1.1: BlobToolKit cumulative scaffold plot. The grey line shows cumulative length for all scaffolds. Coloured lines show cumulative lengths of scaffolds assigned to each phylum using the buscogenes taxrule. An interactive version of this figure is available at https://blobtoolkit.genomehubs.org/view/Pandemis%20corylana/dataset/ilPanCory1_1/cumulative.

**Figure 5.**
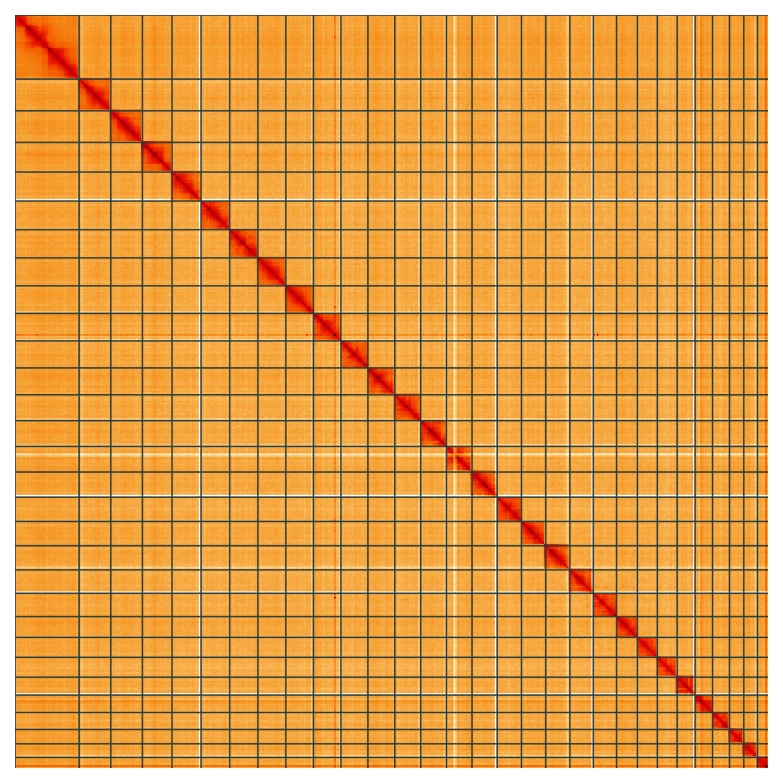
Genome assembly of *Pandemis corylana*, ilPanCory1.1: Hi-C contact map of the ilPanCory1.1 assembly, visualised using HiGlass. Chromosomes are shown in order of size from left to right and top to bottom. An interactive version of this figure may be viewed at https://genome-note-higlass.tol.sanger.ac.uk/l/?d=ZXEiFv_ASrm4DroJa5smuw.

The final assembly has a total length of 441.6 Mb in 39 sequence scaffolds with a scaffold N50 of 15.7 Mb ( Table 1). The snailplot in Figure 2 provides a summary of the assembly statistics, while the distribution of assembly scaffolds on GC proportion and coverage is shown in Figure 3. The cumulative assembly plot in Figure 4 shows curves for subsets of scaffolds assigned to different phyla. Most (99.93%) of the assembly sequence was assigned to 30 chromosomal-level scaffolds, representing 29 autosomes and the Z sex chromosome. Chromosome Z was assigned by synteny to *Pandemis cinnamomeana* (GCA_932294345.1) ( Boyes *et al.*, 2023). Chromosome-scale scaffolds confirmed by the Hi-C data are named in order of size ( Figure 5; Table 2). While not fully phased, the assembly deposited is of one haplotype. Contigs corresponding to the second haplotype have also been deposited. The mitochondrial genome was also assembled and can be found as a contig within the multifasta file of the genome submission.

**Table 1.** Genome data for *Pandemis corylana*, ilPanCory1.1.

Project accession data		
Assembly identifier	ilPanCory1.1	
Species	*Pandemis corylana*	
Specimen	ilPanCory1	
NCBI taxonomy ID	1101029	
BioProject	PRJEB55960	
BioSample ID	SAMEA7701543	
Isolate information	ilPanCory1, male: whole organism (DNA sequencing) ilPanCory2: head and thorax (Hi-C data)	
Assembly metrics *		*Benchmark*
Consensus quality (QV)	65.2	*≥ 50*
*k*-mer completeness	100%	*≥ 95%*
BUSCO **	C:98.5%[S:98.0%,D:0.5%],F:0.4%,M:1.1%,n:5,286	*C ≥ 95%*
Percentage of assembly mapped to chromosomes	99.93%	*≥ 95%*
Sex chromosomes	Z chromosome	*localised homologous pairs*
Organelles	Mitochondrial genome assembled	*complete single alleles*
Raw data accessions		
PacificBiosciences SEQUEL II	ERR10224906	
Hi-C Illumina	ERR10802449	
Genome assembly		
Assembly accession	GCA_949127965.1	
*Accession of alternate haplotype*	GCA_949127985.1	
Span (Mb)	441.6	
Number of contigs	75	
Contig N50 length (Mb)	12.1	
Number of scaffolds	39	
Scaffold N50 length (Mb)	15.7	
Longest scaffold (Mb)	37.5	
Genome annotation		
Number of protein-coding genes	19,608	
Number of gene transcripts	19,780	

* Assembly metric benchmarks are adapted from column VGP-2020 of “Table 1: Proposed standards and metrics for defining genome assembly quality” from ( Rhie *et al.*, 2021). ** BUSCO scores based on the lepidoptera_odb10 BUSCO set using v5.3.2. C = complete [S = single copy, D = duplicated], F = fragmented, M = missing, n = number of orthologues in comparison. A full set of BUSCO scores is available at https://blobtoolkit.genomehubs.org/view/Pandemis%20corylana/dataset/ilPanCory1_1/busco.

**Table 2.** Chromosomal pseudomolecules in the genome assembly of *Pandemis corylana*, ilPanCory1.

INSDC accession	Chromosome	Length (Mb)	GC%
OX421851.1	1	18.52	38.5
OX421852.1	2	18.48	39.0
OX421853.1	3	17.32	39.0
OX421854.1	4	16.93	38.5
OX421855.1	5	16.74	38.5
OX421856.1	6	16.5	38.5
OX421857.1	7	16.36	39.0
OX421858.1	8	16.08	38.5
OX421859.1	9	16.08	38.5
OX421860.1	10	15.79	38.5
OX421861.1	11	15.68	39.0
OX421862.1	12	15.1	39.0
OX421863.1	13	15.04	38.5
OX421864.1	14	14.98	39.0
OX421865.1	15	14.69	39.0
OX421866.1	16	14.22	39.0
OX421867.1	17	14.21	39.0
OX421868.1	18	14.08	39.5
OX421869.1	19	13.66	39.0
OX421870.1	20	13.63	39.0
OX421871.1	21	12.14	39.5
OX421872.1	22	11.69	39.0
OX421873.1	23	11.56	39.5
OX421874.1	24	10.46	39.5
OX421875.1	25	10.16	41.0
OX421876.1	26	9.94	39.5
OX421877.1	27	8.4	40.0
OX421878.1	28	7.98	41.0
OX421879.1	29	7.33	41.0
OX421850.1	Z	37.48	38.5
OX421880.1	MT	0.02	20.0

The estimated Quality Value (QV) of the final assembly is 65.2 with *k*-mer completeness of 100%, and the assembly has a BUSCO v5.3.2 completeness of 98.5% (single = 98.0%, duplicated = 0.5%), using the lepidoptera_odb10 reference set ( *n* = 5,286).

Metadata for specimens, barcode results, spectra estimates, sequencing runs, contaminants and pre-curation assembly statistics are given at https://links.tol.sanger.ac.uk/species/1101029.

## Genome annotation report

The *Pandemis corylana* genome assembly (GCA_949127965.1) was annotated using the Ensembl rapid annotation pipeline ( Table 1; https://rapid.ensembl.org/Pandemis_corylana_GCA_949127965.1/Info/Index). The resulting annotation includes 19,780 transcribed mRNAs from 19,608 protein-coding genes.

## Methods

### Sample acquisition and nucleic acid extraction

A male *Pandemis corylana* (specimen ID Ox000682, ToLID ilPanCory1) was collected from Wytham Woods, Oxfordshire (biological vice-country Berkshire), UK (latitude 51.77, longitude –1.34) on 2020-07-20 using a light trap. The specimen was collected and identified by Douglas Boyes (University of Oxford) and preserved on dry ice.

The specimen used for Hi-C sequencing (specimen ID NHMUK013805966, ToLID ilPanCory2) was collected from Hartslock Nature Reserve, England, UK (latitude 51.51, longitude –1.11) on 2021-07-29 using a light trap. The specimen was collected by Ian Sims and identified by Ian Sims and David Lees (Natural History Museum) and preserved on dry ice.

High molecular weight (HMW) DNA was extracted at the Tree of Life laboratory, Wellcome Sanger Institute (WSI), following a sequence of core procedures: sample preparation; sample homogenisation; HMW DNA extraction; DNA fragmentation; and DNA clean-up. The ilPanCory1 sample was weighed and dissected on dry ice ( Jay *et al.*, 2023). The sample was homogenised using a Nippi Powermasher fitted with a BioMasher pestle ( Denton *et al.*, 2023a). HMW DNA was extracted using the Automated MagAttract v1 protocol ( Sheerin *et al.*, 2023). HMW DNA was sheared into an average fragment size of 12–20 kb in a Megaruptor 3 system with speed setting 30 ( Todorovic *et al.*, 2023). Sheared DNA was purified by solid-phase reversible immobilisation ( Strickland *et al.*, 2023): in brief, the method employs a 1.8X ratio of AMPure PB beads to sample to eliminate shorter fragments and concentrate the DNA. The concentration of the sheared and purified DNA was assessed using a Nanodrop spectrophotometer and Qubit Fluorometer and Qubit dsDNA High Sensitivity Assay kit. Fragment size distribution was evaluated by running the sample on the FemtoPulse system.

Protocols employed by the Tree of Life laboratory are publicly available on protocols.io ( Denton *et al.*, 2023b).

### Sequencing

Pacific Biosciences HiFi circular consensus DNA sequencing libraries were constructed according to the manufacturers’ instructions. DNA sequencing was performed by the Scientific Operations core at the WSI on a Pacific Biosciences SEQUEL II instrument. Hi-C data were also generated from head and thorax tissue of ilPanCory2 using the Arima2 kit and sequenced on the Illumina NovaSeq 6000 instrument.

### Genome assembly, curation and evaluation

Assembly was carried out with Hifiasm ( Cheng *et al.*, 2021) and haplotypic duplication was identified and removed with purge_dups ( Guan *et al.*, 2020). The assembly was then scaffolded with Hi-C data ( Rao *et al.*, 2014) using YaHS ( Zhou *et al.*, 2023). The assembly was checked for contamination and corrected as described previously ( Howe *et al.*, 2021). Manual curation was performed using HiGlass ( Kerpedjiev *et al.*, 2018) and Pretext ( Harry, 2022). The mitochondrial genome was assembled using MitoHiFi ( Uliano-Silva *et al.*, 2023), which runs MitoFinder ( Allio *et al.*, 2020) or MITOS ( Bernt *et al.*, 2013) and uses these annotations to select the final mitochondrial contig and to ensure the general quality of the sequence.

A Hi-C map for the final assembly was produced using bwa-mem2 ( Vasimuddin *et al.*, 2019) in the Cooler file format ( Abdennur & Mirny, 2020). To assess the assembly metrics, the *k*-mer completeness and QV consensus quality values were calculated in Merqury ( Rhie *et al.*, 2020). This work was done using Nextflow ( Di Tommaso *et al.*, 2017) DSL2 pipelines “sanger-tol/readmapping” ( Surana *et al.*, 2023a) and “sanger-tol/genomenote” ( Surana *et al.*, 2023b). The genome was analysed within the BlobToolKit environment ( Challis *et al.*, 2020) and BUSCO scores ( Manni *et al.*, 2021; Simão *et al.*, 2015) were calculated.

Table 3 contains a list of relevant software tool versions and sources.

**Table 3.** Software tools: versions and sources.

Software tool	Version	Source
BlobToolKit	4.2.1	https://github.com/blobtoolkit/blobtoolkit
BUSCO	5.3.2	https://gitlab.com/ezlab/busco
Hifiasm	0.16.1-r375	https://github.com/chhylp123/hifiasm
HiGlass	1.11.6	https://github.com/higlass/higlass
Merqury	MerquryFK	https://github.com/thegenemyers/MERQURY.FK
MitoHiFi	2	https://github.com/marcelauliano/MitoHiFi
PretextView	0.2	https://github.com/wtsi-hpag/PretextView
purge_dups	1.2.3	https://github.com/dfguan/purge_dups
sanger-tol/genomenote	v1.0	https://github.com/sanger-tol/genomenote
sanger-tol/readmapping	1.1.0	https://github.com/sanger-tol/readmapping/tree/1.1.0
YaHS	1.2a	https://github.com/c-zhou/yahs

### Genome annotation

The BRAKER2 pipeline ( Brůna *et al.*, 2021) was used in the default protein mode to generate annotation for the *Pandemis corylana* assembly (GCA_949127965.1) in Ensembl Rapid Release.

### Wellcome Sanger Institute – Legal and Governance

The materials that have contributed to this genome note have been supplied by a Darwin Tree of Life Partner. The submission of materials by a Darwin Tree of Life Partner is subject to the **‘Darwin Tree of Life Project Sampling Code of Practice’**, which can be found in full on the Darwin Tree of Life website here. By agreeing with and signing up to the Sampling Code of Practice, the Darwin Tree of Life Partner agrees they will meet the legal and ethical requirements and standards set out within this document in respect of all samples acquired for, and supplied to, the Darwin Tree of Life Project. 

Further, the Wellcome Sanger Institute employs a process whereby due diligence is carried out proportionate to the nature of the materials themselves, and the circumstances under which they have been/are to be collected and provided for use. The purpose of this is to address and mitigate any potential legal and/or ethical implications of receipt and use of the materials as part of the research project, and to ensure that in doing so we align with best practice wherever possible. The overarching areas of consideration are:

•   Ethical review of provenance and sourcing of the material

•   Legality of collection, transfer and use (national and international) 

Each transfer of samples is further undertaken according to a Research Collaboration Agreement or Material Transfer Agreement entered into by the Darwin Tree of Life Partner, Genome Research Limited (operating as the Wellcome Sanger Institute), and in some circumstances other Darwin Tree of Life collaborators.

## Data Availability

European Nucleotide Archive: *Pandemis corylana* (chequered fruit-tree tortrix). Accession number PRJEB55960; https://identifiers.org/ena.embl/PRJEB55960 ( Wellcome Sanger Institute, 2022). The genome sequence is released openly for reuse. The *Pandemis corylana* genome sequencing initiative is part of the Darwin Tree of Life (DToL) project. All raw sequence data and the assembly have been deposited in INSDC databases. Raw data and assembly accession identifiers are reported in Table 1.

## Author information

Members of the University of Oxford and Wytham Woods Genome Acquisition Lab are listed here: https://doi.org/10.5281/zenodo.7125292.

Members of the Natural History Museum Genome Acquisition Lab are listed here: https://doi.org/10.5281/zenodo.7139035.

Members of the Darwin Tree of Life Barcoding collective are listed here: https://doi.org/10.5281/zenodo.4893703.

Members of the Wellcome Sanger Institute Tree of Life Management, Samples and Laboratory team are listed here: https://doi.org/10.5281/zenodo.10066175.

Members of Wellcome Sanger Institute Scientific Operations: Sequencing Operations are listed here: https://doi.org/10.5281/zenodo.10043364.

Members of the Wellcome Sanger Institute Tree of Life Core Informatics team are listed here: https://doi.org/10.5281/zenodo.10066637.

Members of the Tree of Life Core Informatics collective are listed here: https://doi.org/10.5281/zenodo.5013541.

Members of the Darwin Tree of Life Consortium are listed here: https://doi.org/10.5281/zenodo.4783558.
