# Supplementary material for: The genome sequence of the Chequered Fruit-tree Tortrix, Pandemis corylana (Fabricius, 1794)
Source: Wellcome Open Res. 2024 Jan 8;9:6. [Version 1] doi: 10.12688/wellcomeopenres.20572.1 (PMC10891431; doi:10.12688/wellcomeopenres.20572.1)
Supplement: Peer review history

# Reviewer report: Reviewer response for version 1

**DOI:** 10.21956/wellcomeopenres.22771.r73310

**Published:** 2024-02-16

**Authors:** Kuppusamy Sivasankaran

**Affiliations:** 1 Entomology Research Institute, Loyola Collège, Chennai, India

I appreciate the authors for assembling the genome sequence of Chequered Fruit-tree Tortrix *Pandemis corylana* (Fabricius, 1794). Using 39 scaffolds, the authors sequenced approximately 441.6 mega bases of the genome assembly. The authors have accurately submitted the sequence to the open database. These robust sequence data will be useful to phylogenomic researchers.  

**Suggestion**

 The authors have given the mitogenome sequence length in the abstract, but they haven’t given in the manuscript. It can be included in the manuscript.

Are sufficient details of methods and materials provided to allow replication by others?

Yes

Is the rationale for creating the dataset(s) clearly described?

Yes

Are the datasets clearly presented in a useable and accessible format?

Yes

Are the protocols appropriate and is the work technically sound?

Yes

Reviewer Expertise:

Phylogenetic analysis of Moths

I confirm that I have read this submission and believe that I have an appropriate level of expertise to confirm that it is of an acceptable scientific standard.

# Reviewer report: Reviewer response for version 1

**DOI:** 10.21956/wellcomeopenres.22771.r73306

**Published:** 2024-02-16

**Authors:** Tyler Scott Alioto

**Affiliations:** 1 Barcelona Institute of Science and Technology (BIST), Baldiri i Reixac 4, 08028 Barcelona, Spain

This genome note reports the specimen collection, sequencing, genome assembly and annotation of the chequered fruit-tree tortrix, Pandemis corylana. The genome note is complete, well-organized and well-written. Standard state-of-the-art methods were used to achieve a high-quality chromosome-level primary assembly (as well as the mitogenome, alternate haplotypes and a Wolbachia symbiont) that meet the minimum EBP reference genome standards. All data has been deposited in the INSDC and protocols, quality control info (tolqc), and analyses have been made public.

Are sufficient details of methods and materials provided to allow replication by others?

Yes

Is the rationale for creating the dataset(s) clearly described?

Yes

Are the datasets clearly presented in a useable and accessible format?

Yes

Are the protocols appropriate and is the work technically sound?

Yes

Reviewer Expertise:

Genome assembly, genome annotation, gene prediction

I confirm that I have read this submission and believe that I have an appropriate level of expertise to confirm that it is of an acceptable scientific standard.
